# Untreated depressive symptoms significantly worsen quality of life in old age and may lead to the misdiagnosis of dementia: a cross-sectional study

**DOI:** 10.1186/s12991-020-00302-6

**Published:** 2020-09-15

**Authors:** Viktor Voros, Sandor Fekete, Tamas Tenyi, Zoltan Rihmer, Ilona Szili, Peter Osvath

**Affiliations:** 1 Department of Psychiatry and Psychotherapy, Faculty of Medicine, University of Pecs, Pecs, Hungary; 2 Department of Psychiatry and Psychotherapy, Semmelweis University, Faculty of Medicine, Budapest, Hungary; 3 Laboratory for Suicide Research and Prevention, National Institute of Psychiatry and Addictions, Budapest, Hungary; 4 Department of Clinical Psychology, Karoli Gaspar University of the Reformed Church, Budapest, Hungary

**Keywords:** Quality of Life (QoL), Old age, Depression, Dementia, Pseudo-dementia

## Abstract

**Background:**

Several studies demonstrated the role of depressive mood and cognitive impairment in the background of elevated mortality and decreased Quality of Life (QoL) in old age. Our aim was to assess depressive and cognitive symptoms among older people in order to determine if those are recognized and treated or not, to elucidate the association between them and to investigate their impact on QoL.

**Methods:**

In the framework of the ICT4Life project self-administered questionnaires and clinical screening tools were used to assess QoL, depressive symptoms and cognitive functions of 60 older persons over the age of 65.

**Results:**

Males found to be depressed (53.8 vs. 40.4%) and cognitively declined (53.8 vs. 48.9%) more frequently; and had higher scores on the depression (6.85 vs. 5.32) and lower on the QoL (47.38 vs. 50.19) scales. Depressed older persons had lower cognitive levels (24.39 vs. 21.52) and their QoL was significantly poorer (53.97 vs. 43.85) than that of the non-depressed subjects. Depressive symptoms were detected in almost half of the older adults (43.9%), and the majority (80.77%) did not receive antidepressant medication.

**Conclusions:**

Depressive and cognitive symptoms found to be common among older people, but were not recognized and treated in most cases. Close correlation between depression and cognitive impairment was also confirmed, as well as the key role of depression in the background of pseudo-dementia and QoL decline. Early recognition of depressive symptoms is important not only to treat the underlying mood disorder, but also to improve QoL of older persons.

## Background

In our aging society, not only the maintenance of health of old people, but also the restoration of their Quality of Life (QoL) has become an important goal. Therefore, many studies aimed to detect factors influencing QoL, as those may have preventive and therapeutic significance. Several studies have demonstrated the role of depressive disorders and cognitive impairment in the background of elevated mortality and decreasing QoL in old age [1–4].

The close correlation between depression and dementia is well-known, however the link between them is still unclear, and a subject of intensive research. According to a recent meta-analysis, depression in old age can be an important risk factor for several types of dementia [5]. Furthermore, it was suggested that the same risk factors may be present in the background of dementia and depression in elderly, or even the same pathophysiological impairments may lead to both conditions [6–8]. Based on these findings, depression may be an early marker of mild cognitive impairment and incipient dementia [9]. Furthermore, depression and cognitive impairments are also stressed as important risk factors for suicide in older adults [10].

Based on the above, we investigated depressive symptoms, cognitive functions and QoL of older people, and we also assessed the occurrence of the diagnosed and treated mental disorders and psychotropic medications. The primary goal of our study was to assess depressive and cognitive symptoms among older people in order to determine if those are recognized and effectively treated or not. We also aimed to better elucidate the association between depressive and cognitive symptoms and to investigate their impact on QoL. Based on the literature, our hypothesis was that in many cases cognitive decline and mood disorders are not recognized and treated, and these both negatively affect the QoL of older persons [11, 12].

## Methods

In the framework of the ICT4Life project, self-administered questionnaires and clinical screening tools were used to assess QoL, depressive symptoms and cognitive functions of 60 older persons (47 females and l3 males) over the age of 65, who live independently in their own apartments and participate in the daily care facility programme for older people of the Integrated Social Institution in Pecs, Hungary [13]. Participation was voluntary and anonymous following the signature of the informed consent form (ICF). The study was conducted in 2017–2018, the data collection and medical record review lasted for 6 months. The questionnaires were administered by trained clinical psychologists, the clinical assessments and the medical record review were completed by psychiatrists. The study was approved by the local Ethics Committee.

Two tests were used to assess QoL of the participants, the abbreviated version of the Older People Quality of Life (OPQOL-Brief) and the Quality of Life in Alzheimer's Disease (QOL-AD) questionnaires. The former is a 13-item short version of the well-known QoL scale (OPQOL-35), which has beneficial psychometric properties [14]. The QOL-AD questionnaire was specifically developed to assess the QoL of people with dementia, especially Alzheimer’s disease. It is based on a semi-structured interview, in which 13 items regarding QoL are covered. Evaluation of the questions is based on a four-grade Likert scale ranging from bad to excellent [15]. The Mini-Mental State Examiner (MMSE) was used to evaluate the cognitive levels of the subjects. The total score is 30 points with a 24 points of cut-off score for dementia [16]. Depressive symptoms and the severity of depression was measured by the 15-item Geriatric Depression Scale (GDS) [17]. The short version of the GDS can be easily applied to patients even with mild or moderate cognitive decline as well; and is considered to be a useful tool to differentiate depressed older persons from the non-depressed ones [18].

We also assessed the socio-demographic characteristics, the physical and mental disorders, and medication usage with semi-structured interviews. The review of medical records was also completed in order to control data captured during the semi-structured interviews.

The data were analysed by using SPSS 20.0. We performed descriptive statistical analyses,* χ*^2^-probe and Mantel–Haenszel test were used to identify group differences, and ANOVA for comparison of mean values. The level of significance was set at *p* < 0.05.

## Results

### Clinical characteristics of the study population and gender differences

The *study population* comprised 60 older adults over the age of 65 years (13 males, 47 females), their age ranged from 65 to 93 years (mean age: 77.12 years).

Regarding *gender differences*, the mean age of males was lower than that of females (74.08 vs. 77.96 years). Common diseases (such as hypertension, rheumatological problems, cardiac diseases, arteriosclerosis, etc.) characterized a significant proportion of both older males and females, as did other physical comorbidities. Both females and males took a number of medications (from 0 to 24 medications/participant, mean: 6.13) because of their several illnesses. In addition to medications taken for the underlying physical conditions (such as antihypertensives, other cardiac medications, anticoagulants, and gastrointestinal drugs), the use of analgesics and psychotropic medications was also significant (Table 1). Males had more previous (46.2% vs. 36.2%) and current (30.8% vs. 23.4%) psychiatric treatments. However, no significant gender differences were found in the occurrence of the already diagnosed mental disorders, in the number of previous in-patient or out-patient psychiatric treatments, or in the current use of psychiatric medications (Table 1). Among mental disorders in the patients’ history, addictions were more common among males (15.4% vs. 0%), while depression (21.3% vs. 15.4%) and dementia (21.3% vs. 7.7%) in females.

**Table 1:** The main clinical characteristics and gender differences in the old age study population (*N* = 60) (Pearson’s Chi-square test and Mantel–Haenszel test)

	Male *N* = 13 (%)	Female *N* = 47 (%)	Total *N* = 60 (%)	Chi^2^	*p*-value	Mantel–Haenszel test (Male = 1)	
						OR	*p*-value
Dementia (MMSE)	7 (53.8)	23 (48.9)	30 (50)	0.001	0.617	0.986	0.982
Depression (GDS)	7 (53.8)	19 (40.4)	26 (43.3)	0.74	0.29	0.582	0.39
Psychiatric treatment (previous)	6 (46.2)	17 (36.2)	23 (38.3)	0.429	0.365	0.661	0.514
Psychiatric treatment (current)	4 (30.8)	11 (23.4)	15 (25)	0.295	0.415	0.688	0.589
Mental disorder (patient’s history)							
Depression	2 (15.4)	10 (21.3)	12 (20)	0.221	0.488	1.486	0.64
Dementia	1 (7.7)	10 (21.3)	11 (18.3)	1.255	0.247	3.243	0.285
Addiction	2 (15.4)	0 (0)	2 (3.3)	7.48	0.44	–	–
Psychosis	1 (7.7)	3 (6.4)	4 (6.7)	0.028	0.634	0.818	0.867
Psychopharmacologic medication (current)	7 (53.8)	25 (53.2)	32 (53.3)	0.002	0.608	0.974	0.967
Anxiolytic	2 (15.4)	11 (23.4)	13 (21.7)	0.386	0.422	1.618	0.538
Hypnotic	1 (7.7)	3 (6.4)	4 (6.7)	0.028	0.634	0.818	0.867
Antidepressant	3 (23.1)	8 (17)	11 (18.3)	0.249	0.443	0.684	0.619
Antipsychotic	3 (23.1)	6 (12.8)	9 (15)	0.849	0.299	0.488	0.364

*MMSE* Mini-Mental State Examiner, *GDS* Geriatric Depression Scale, OR odds ratio

About half of the older adults took *psychotropic medications* (mostly anxiolytics) currently (males: 53.8%, females: 53.2%). Although there was no significant difference between males and females in the occurrence of previous psychotic disorders (7.7% vs. 6.4%) and depression (15.4% vs. 21.3%), males were more likely to take antipsychotics (23.1% vs. 12.8%) and antidepressants (23.1% vs. 17%); and less likely benzodiazepines (15.4% vs. 23.4%) (Table 1).

### Assessment of depressive and cognitive symptoms in the study population

Based on current assessments, the *cognitive test (MMSE)* detected cognitive impairment reaching the level of dementia in half of the older adults (males: 53.8%, females: 48.9%), while more than 40% of them (males: 53.8%, females: 40.4%) had depression according to the *depression screening tool (GDS)* (Table 1). Although there was no significant gender difference in the mean values of the individual scales (MMSE, GDS, and QoL), males were found to be depressed and cognitively declined more frequently, and had higher scores on the depression scale (6.85 vs. 5.32) and lower on the QoL scales (OPQOL-Brief: 47.38 vs. 50.19; QOL-AD: 29.27 vs. 32.1).

*Subjects, who were screened to be depressed with the GDS* were more likely to be treated either in the past (57.7% vs. 23.5%) or currently (34.6% vs. 17.6%), and they were also taking psychiatric medications much more frequently (73.1% vs. 38.2%) than those without depression (Table 2). However, there was no significant difference in the use of benzodiazepines (23.1% vs. 20.6%) and antidepressants (19.2% vs. 17.6%), only antipsychotics were taken more frequently (34.6% vs. 0%) by depressed elderly. Previously diagnosed mental disorders (depression, dementia, psychosis) were also more common in the group who were currently screened to be positive for depression, but the differences were not significant, not even for depression (depression: 26.9% vs. 14.7%; dementia: 23.1% vs. 14.7%; psychotic disorders: 11.5% vs. 2.9%), which may be also due to the low number of positive cases. However, cognitive decline reaching the level of dementia, as measured with the MMSE was more frequent among currently depressed—measured with GDS—subjects (61.5% vs 41.2%) (Table 2). In the group of older persons screened to be positive for depression, the mean scores of the cognitive (MMSE) and the QoL scales were significantly lower (Table 3).

**Table 2:** The main clinical characteristics of the study population (*N* = 60) of older adults with and without current depression according to the Geriatric Depression Scale (Pearson’s Chi-square test and Mantel–Haenszel test)

	Non-depressed *N* = 34 (%)	Depressed *N* = 26 (%)	Total *N* = 60 (%)	Chi^2^	*p*-value	Mantel–Haenszel test (non-depressed = 1)	
						OR	*p*-value
Dementia (MMSE)	14 (41.2)	16 (61.5)	30 (50)	4.014	0.041	3.102	0.049*
Psychiatric treatment (previous)	8 (23.5)	15 (57.7)	23 (38.3)	7.274	0.007	4.43	0.009*
Psychiatric treatment (current)	6 (17.6)	9 (34.6)	15 (25)	2.262	0.115	2.471	0.138
Mental disorder (patient’s history)							
Depression	5 (14.7)	7 (26.9)	12 (20)	1.374	0.198	2.137	0.247
Dementia	5 (14.7)	6 (23.1)	11 (18.3)	0.69	0.309	1.74	0.41
Psychosis	1 (2.9)	3 (11.5)	4 (6.7)	1.75	0.212	4.304	0.219
Psychopharmacologic medication (current)	13 (38.2)	19 (73.1)	32 (53.3)	7.186	0.007	4.385	0.009*
Benzodiazepine	7 (20.6)	6 (23.1)	13 (21.7)	0.054	0.53	1.157	0.817
Antidepressant	6 (17.6)	5 (19.2)	11 (18.3)	0.025	0.567	1.111	0.875
Antipsychotic	0 (0)	9 (34.6)	9 (15)	13.846	0	–	–

*MMSE* Mini-Mental State Examiner, *OR* odds ratio

* Significant (*p* < 0.05)

**Table 3:** Mean scores of the different clinical scales and screening tools completed by the older adults (*N* = 60) with and without current depression according to the Geriatric Depression Scale (ANOVA test)

	Non-depressed	SD	Depressed	SD	Total	SD	*F*	*p*-value
Age	76.35	9.09	78.12	6.26	77.12	7.98	0.716	0.401
MMSE	24.39	5	21.52	5.02	23.21	5.17	4.454	0.039*
GDS	2.79	1.59	9.38	2.73	5.65	3.93	137.614	0*
OPQOL-Brief	53.97	5.86	43.85	8.8	49.58	8.81	28.547	0*
QUOL-AD	34.07	4.8	28	5.34	31.42	5.85	17.137	0*

*MMSE* Mini-Mental State Examiner, *GDS* Geriatric Depression Scale, *OPQOL-Brief* Older People Quality of Life scale brief version, *QOL-AD* Quality of Life in Alzheimer's Disease scale, *SD* standard deviation

* Significant (*p* < 0.05)

## Discussion

*According to our findings*, depressive and cognitive symptoms are common among older people, and these symptoms are not recognized and treated in many cases, as hypothesized. Furthermore, older adults with depressive symptoms have lower cognitive functions and QoL. The significance of our research is emphasized by the fact that the early detection and adequate management of affective symptoms in old age may contribute to the improvement of mental disorders and QoL.

As *limitations of the study*, first we highlight the low number of cases and the fact that only those older persons took part in the assessments, who signed the ICF, and whose mental and physical condition was good enough to be able to consent and complete the questionnaires and screening tests. However, our results can be considered representative of this population living independently in their own apartments and has regular contact with the daily care centre for older people. Secondly, the clinical screening tools for assessing depressive and cognitive symptoms completed in the study cannot be used to clinically diagnose depression or dementia. Therefore, the finding of significant proportion of depressive symptoms does not necessarily mean that these older persons suffered from clinical major depressive disorder (MDD). However, it is important to highlight that although most of these older persons were in regular contact with health care systems due to their physical illnesses, their depressive symptoms were mostly unrecognized, thus specific psychiatric assessment and adequate treatment could not be performed. Lastly, the use of structured clinical interview may be of limited value in older adults because of the frequent occurrence of cognitive decline. Therefore, extensive review of medical records was also completed.

About *half of the subjects* took some psychotropic medications, and the same proportion had cognitive impairment reaching the level of dementia (mainly mild, according to the MMSE), and a little less (but more than 40%) found to be depressed by the GDS depression screening tool (Table 1). Although it was somewhat more common to take antidepressant medications, there was a greater proportion of current depressive symptoms among males. No other major differences were found between *genders*.

Among the participants, out of the 11 patients taking *antidepressants* 6 patients were without current depressive symptoms, which may indicate the efficacy of the antidepressant treatment in old age (Table 2). 26 participants had current depressive symptoms, but only 9 of them contacted a psychiatrist, and 7 of them had a clinical diagnosis of depression. Furthermore, only 5 of them received antidepressant medication (which also means that 21 older adults were found to have depressive symptoms, but their depression was not treated), but still they had some depressive symptoms. On the other hand, these patients with current depressive symptoms were more likely to receive *antipsychotic* medications. This may indicate that only certain symptoms of the depressive disorder (sleep disturbance, anxiety, agitation) have been recognized and treated (with antipsychotics), while the underlying mood disorder was not considered, and therefore an adequate antidepressant treatment was lacking. Theoretically, antipsychotics, especially first-generation ones may be associated with the occurrence of depressive symptoms, but all the patients in the study population were treated with second-generation antipsychotics, which rarely induce depressive symptoms. Moreover, as it is known from the literature and clinical practice, some second-generation antipsychotics may improve depressive symptoms or can even work as antidepressants [19]. So, these antipsychotics might be prescribed to treat depression in older persons. Although for many patients there was only partial response to these antipsychotic or antidepressant medications, as—at least some of them—depressive symptoms were still present.

Based on our analysis, among *older adults with current depression* (according to the GDS) cognitive decline (assessed with the MMSE) was significantly more frequent and more pronounced, but this was not recognized and treated. In line with previous literature data, the results of this study showed that depressed (according to the GDS) older persons had lower cognitive levels and their QoL was also significantly poorer than that of the non-depressed. Our results also confirm close correlation between depression and cognitive impairment, as well as the key role of depression in the background of QoL decline [3, 20, 21]. On the other hand, it is well-known that reversible depression-related cognitive decline (also known as depressive pseudo-dementia) is often considered as true dementia and these patients do not receive adequate treatment for their mood disorder [22]. However, the depressive pseudo-dementia in old age seems to be a long-term predictor of true dementia [5, 23].

## Conclusions

The common depressive symptoms in older adults contributes to poorer QoL and may also lead to the misdiagnosis of dementia. Our research also refers to the problems of recognition and effective treatment of depression in old age, which can be further complicated by the also common symptoms of cognitive impairment among depressive patients (depressive pseudo-dementia) [7, 24]. As depressive symptoms may be the first signs of dementia [6], early recognition of depression is important not only to treat the mood disorder, but also for the early detection and treatment of cognitive impairment [5]. The preventive significance of our results is evidenced by the fact that the screening, treatment and follow-up of depressed older patients is of high importance not only to adequately treat the mood disorder and avoid the misdiagnosis of depressive-pseudodementia as true dementia, but also to improve the QoL of the older persons. Furthermore, it may be beneficial to prevent further cognitive decline [9, 22] and other complications, such as increased cardiovascular morbidity/mortality and suicide [10, 25].

## Data Availability

The datasets used and/or analysed during the current study are available from the corresponding author on reasonable request.

## Abbreviations

GDS: Geriatric Depression Scale
ICF: Informed Consent Form
ICT4Life project: Information and Communication Technology for Life project
MDD: Major depressive disorder
MMSE: Mini-Mental State Examiner
OPQOL: Older People Quality of Life
QoL: Quality of Life
QOL-AD: Quality of Life in Alzheimer's Disease
